# Behind Protein Synthesis: Amino Acids—Metabokine Regulators of Both Systemic and Cellular Metabolism

**DOI:** 10.3390/nu15132892

**Published:** 2023-06-26

**Authors:** Evasio Pasini, Giovanni Corsetti, Francesco S. Dioguardi

**Affiliations:** 1 Italian Association of Functional Medicine, 20855 Milan, Italy; 2 Department of Clinical and Experimental Sciences, University of Brescia, 25123 Brescia, Italy; giovanni.corsetti@unibs.it; 3 Department of Internal Medicine, University of Cagliari, 09042 Cagliari, Italy; fsdioguardi@gmail.com

Recent scientific research suggests that amino acids (AA) are not only the “building bricks” of protein synthesis but may also be considered “metabokines”. Indeed, emerging evidence shows that certain metabolites and nutrients (including AA), which are not vitamins, cytokines and/or hormones, are able to regulate fundamental metabolic cell pathways. These bioactive metabolites are called metabokines [1].

This Special Issue reinforces the concept that AA are able to influence the cell metabolism of both healthy and diseased cells. Moreover, the articles we have included here show that specific mixtures of AA could influence neoplastic cells and tumour-related disorders.

Jiménez-Alonso and colleagues showed that an artificial diet based on a mixture of selective AA increased the life expectancy of mice injected with CT26 WT murine colon cancer cells. This finding would suggest that this specific mixture of AA may have therapeutic potential for colon cancer [2]. Additionally, Llop-Hernández’s article investigated a very interesting aspect of an innovative and integrated therapy for carcer treatment. It showed that specific patterns of various carbon/nitrogen energy sources (including certain AA) can force tumoral cells into a non-proliferative senescence state [3].

A cutting-edge paper by Beaudry and collaborators reviewed the mechanisms of leucine supplementation used to counteract cancer-induced cachexia [4]. In addition, we can note that blood AA levels are prognostic markers in patients with cancer. Indeed, low levels of circulating histidine, leucine and phenylalanine, concomitant with reduced anthropometric measurements and severe inflammation, has been found by Yeh and collaborators to correlate with pre-treatment Glasgow prognostic scores in patients with squamous cell carcinoma. As a result, these authors suggest that the assessment of nutritional, anthropometric and inflammatory status should be included in the enrolment criteria in future studies that analyse the effects of anti-tumoral drags [5].

Interestingly, the paper from Corsetti and co-workers shows that a specific mixture of free essential-AA (EAA), formulated according to metabolic human needs, reduces the cardiotoxicity induced by doxorubicin in mice, providing a theoretical basis for EAA administration in alleviating chemotherapy-related damages in clinical practice [6].

The effects of AA-mediated regulation of both lipidic and glucose metabolism has also been shown by several articles in this Special Issue. The effects of sulphuric-containing-AA (SCAA) and branched-chain AA (BCAA) supplementation on lipid and glucose metabolism was investigated in HepG2 cells. The results showed that SCAA and its metabolite (SAMe) reduces hepatic lipogenesis. Moreover, the authors provide evidence that SCAA and BCAA influenced the mRNA expression of lipogenic enzymes and PPAR-y expression, suggesting that AAs could have epigenetic control of gene expression [7]. Corsetti and colleagues demonstrated the effects on body adiposity in mice of an essential amino acids-rich diet (EAARD). They showed that EAARD causes a massive reduction of white adipose tissue and increase of muscle mass. It also promotes thermogenesis, thus increasing the synthesis of UCP-1 and SIRT-3 in brown adipose tissue [8].

The potential role of L-arginine in the prevention and treatment of disturbed carbohydrate and lipid metabolism was extensively reviewed by Szlas and collaborators [9]. AA tryptophan-mediated metabolic regulation has been also demonstrated through host-microbiota interaction. Notably, Jiang and co-workers showed that tryptophan influences the production of specific metabolites and the expression of tight junction proteins, which regulate the gut barrier’s function, thus inhibiting the penetration of toxic factors. In addition, tryptophan–microbiota interaction modulates the immune system, exerting anti-inflammatory and antioxidant effects [10].

AA are also able to influence the metabolism of the nervous system. The effects of D-serine and D-aspartate on the healthy central nervous system and their role in the pathogenesis of schizophrenia are extensively discussed in a review by Nasyrova and collaborators [11].

An especially interesting article of this Special Issue shows that AA-derived compounds might well act as metabokins. It is well known that ammonia is produced by the metabolism of amino acids and other compounds which contain nitrogen. The increased catabolism of proteins causes hyper-ammonaemia, which is a metabolic disturbance responsible for severe neurological damage. The paper by Bélanger-Quintana and colleagues points out the importance of frequent monitoring to assess the presence of hyper-ammonaemia in paediatric and adult patients, to evaluate both the presence of metabolic disturbance and the effects of therapeutical strategies including haemodialysis whenever the blood’s ammonia levels are too high [12].

The clinical management of children with disorders of AA metabolism has also been studied and discussed in this Special Issue. The paper by Lim et al. provides important suggestions for the implementation of feasible and effective strategies to improve and motivate dietary adherence of prescribed diets among both patients and care-givers [13].

In conclusion, the articles published here confirms that AAs and their metabolites could be capable of influencing cellular metabolism and intra-organs/systems crosstalk by acting as metabokines. Notably, it has been reported that certain AAs are capable of also performing an epigenetic control of metabolic pathways of both normal and pathological cells, including neoplastic cells. This evidence provides new knowledge which, if confirmed, would open up new research scenarios regarding the therapeutic use of AAs.
